# Natural Products Counteracting Cardiotoxicity during Cancer Chemotherapy: The Special Case of Doxorubicin, a Comprehensive Review

**DOI:** 10.3390/ijms221810037

**Published:** 2021-09-17

**Authors:** Izabela Koss-Mikołajczyk, Vanja Todorovic, Sladjana Sobajic, Jamal Mahajna, Marko Gerić, Josep A. Tur, Agnieszka Bartoszek

**Affiliations:** 1 Department of Food Chemistry, Technology and Biotechnology, Gdańsk University of Technology, 11/12 Narutowicza St., 80-233 Gdańsk, Poland; agnieszka.bartoszek@pg.edu.pl; 2 Department of Bromatology, Faculty of Pharmacy, University of Belgrade, Vojvode Stepe 450, 11221 Belgrade, Serbia; vanja.todorovic@pharmacy.bg.ac.rs (V.T.); sladjana.sobajic@pharmacy.bg.ac.rs (S.S.); 3 Department of Nutrition and Natural Products, Migal-Galilee Research Institute, Kiryat Shmona 11016, Israel; jamalm@migal.org.il; 4 Department of Nutritional Sciences, Tel-Hai College, Qiryat Shemona 1220800, Israel; 5 Mutagenesis Unit, Institute for Medical Research and Occupational Health, 10000 Zagreb, Croatia; mgeric@imi.hr; 6 Research Group on Community Nutrition & Oxidative Stress, University of Balearic Islands—IUNICS, IDISBA & CIBEROBN (Physiopathology of Obesity and Nutrition), 07122 Palma de Mallorca, Spain; pep.tur@uib.es

**Keywords:** cardiotoxicity, cardioprotectants, phytochemicals, antioxidants, anthracyclines

## Abstract

Cardiotoxicity is a frequent undesirable phenomenon observed during oncological treatment that limits the therapeutic dose of antitumor drugs and thus may decrease the effectiveness of cancer eradication. Almost all antitumor drugs exhibit toxic properties towards cardiac muscle. One of the underlying causes of cardiotoxicity is the stimulation of oxidative stress by chemotherapy. This suggests that an appropriately designed diet or dietary supplements based on edible plants rich in antioxidants could decrease the toxicity of antitumor drugs and diminish the risk of cardiac failure. This comprehensive review compares the cardioprotective efficacy of edible plant extracts and foodborne phytochemicals whose beneficial activity was demonstrated in various models in vivo and in vitro. The studies selected for this review concentrated on a therapy frequently applied in cancer, anthracycline antibiotic—doxorubicin—as the oxidative stress- and cardiotoxicity-inducing agent.

## 1. Introduction

Cancer remains one of the leading health issues worldwide. According to GLOBOCAN, in 2020, there were 19.3 million new cancer cases diagnosed and almost 9.9 million cancer deaths occurred [1]. Compared to 2018, these estimates increased, respectively by about 7% and 3% (18.1 million new cases and 9.6 million deaths in 2018 according to Bray et al. [2]). Cancer also represents an immense economic burden that includes decreased productivity due to lost working hours, costs of prevention, and costs of treatment [3]. Therefore, cancer prevention as well as more efficient treatments are of primary importance.

Modern surgery as an approach to treat cancer was established soon after the discovery of anesthesia in the mid-19th century, while cancer radiotherapy and chemotherapy were first used in the 20th century [4]. By the mid-20th century, a sufficient body of evidence was accumulated to screen the potential anticancer agents, establish proper models to test their efficacy, and convince the scientific community that chemotherapy might become an effective cancer treatment. The second half of the 20th century was marked by several major breakthroughs, such as the use of drugs with different modes of action, the concept of adjuvant therapy, and targeted therapy, with imatinib mesylate—tyrosine kinase inhibitor—being the first FDA-approved targeted anticancer drug [5,6]. With the accumulation of knowledge and constant development in diagnostic, preventive, and treatment regimens, the mortality rates of cancer started to decrease from the 2000s [7]. 

However, despite its unquestionable successes, chemotherapy is still struggling with two major issues: anticancer drug resistance and toxicity [8,9]. Chemotherapy can induce several unpleasant, painful, and severe physical and emotional side symptoms that affect patients’ quality of life [8,9]. Cardiotoxicity, often observed during anticancer chemotherapy, is one of the significant, sometimes substantially delayed, medical complications in cancer survivors after the completion of therapy. The adverse symptoms range from negligible changes in blood pressure and arrhythmias to cardiomyopathy [10]. Significant cardiotoxicity has been observed during chemotherapy with most anti-tumor drugs: mitoxantrone (cardiomyopathy), fluorouracil (cardiac infarction), cyclophosphamide and vinca alkaloids (necrosis of heart cells) [10]. This side effect is particularly dangerous in the case of anthracyclines therapy, especially with doxorubicin (DOX). 

DOX is a very efficient chemotherapeutic agent, the clinical application of which is limited by cumulative, dose-dependent injury of the heart muscle that frequently jeopardizes patients’ life despite successful cancer eradication [11,12]. In a relatively high percentage of patients (reaching over 30% of the population undergoing therapy), congestive cardiac failure is observed following chemotherapy, especially when DOX is administered together with another chemotherapeutics [11]. The cardiotoxicity of anthracyclines, at least to a degree, is associated with their ability to take part in cyclic redox reactions with molecular oxygen. As a by-product of some of these reactions, superoxide anion-radical is formed, which initiates a cascade of reactions involving reactive oxygen (ROS) and reactive nitrogen (RNS) species [13,14,15]. Cardiac tissue is particularly sensitive to oxidative stress. It has been demonstrated that treatment with DOX induced damage of DNA, proteins, lipids, and cellular structures in cardiomyocytes [16,17]. The particularly vulnerable cellular structures that are targets for ROS and RNS in cardiomyocytes are mitochondria [18], myofibrils [19], sarcoplasmic mesh [20], and the nucleus [21,22]. The ultimate effect of such disturbances may be the apoptosis or necrosis of cardiomyocytes [16]. This damage leads to Type I, or permanent cardiotoxicity, in contrast with reversible Type II cardiotoxicity, which is associated with targeted cancer therapies [23].

Therefore, biomarkers (Table 1) that correlate to the level of heart damage are monitored during treatment to control the safe dose of administered cytostatics. This approach makes it possible not only to evaluate the cardiac damage during chemotherapy, but also may be exploited to assess the cardioprotective impact of different substances, including compounds of natural origin (Table 2). One strategy that holds great promise for reducing cardiotoxicity is the use of cardioprotectants or preparations derived from edible plants. Since phytochemicals are both well tolerated and often exhibit high antioxidant activity, they can potentially protect cardiac muscle from the negative effects of oxidative stress.

## 2. Cardioprotective Potential of Foods and Phytochemicals during DOX Chemotherapy

Nutrition is one of the most important factors for maintaining good health, but it also plays a pivotal role in the recovery from diseased state. There is a growing awareness that a better understanding of how to use dietary means to support patients during therapy is urgently needed. Oncological patients are a special case, as medical benefits are often outweighed by the deleterious impact on quality of life [32]. 

This review evaluates fruits, vegetables, herbs, mushrooms, and isolated phytochemicals that display protective properties towards the cardiac muscle during chemotherapy with DOX. It is proposed that the intake of non-toxic purified phytochemicals or preparations based on foods rich in antioxidants could protect oncological patients against undesirable side effects of chemotherapy associated with oxidative stress. Only edible plants and isolated substances for which clinical or pre-clinical studies or in vitro experiments with the use of cardiomyocytes or cell lines derived from the heart tissue of animal embryos were carried out are included in the review. Since the actual numerical data derived from studies employing different experimental models are difficult to compare, we recalculated the published values in the reference to no-treatment-control levels regarded as 100%. In this way, it is easy to assess to what extent DOX treatment impairs the redox homeostasis of cardiomyocytes and how successfully it is restored by treatment with evaluated cardioprotectants.

### 2.1. Cardioprotective Potential of Fruits

#### 2.1.1. Grapes

Epidemiological studies suggest that grapes (*Vitis vinifera* L.) exhibit anti-carcinogenic properties, mostly due to the presence of resveratrol, the most biologically active compound found in these fruits [33,34]. Newer data indicate that resveratrol is also able to protect the hearts of mice against damage induced by DOX during cancer treatment [35,36]. Similarly, grape seed extract (GSE), containing proanthocyanidines, displayed cardioprotective effects during therapy with DOX. This was confirmed by the results of experiments in which mice were treated with DOX (20 mg/kg/day), grape seed extract (100 mg/kg/day orally for 9 days) or DOX in conjunction with grape seed extract (100 mg/kg/day for 7 days before and 2 days after DOX administration). During the study, creatine kinase (CK) activity was measured, whose elevated level is a biomarker of cardiac muscle damage (Table 3). These experiments demonstrated that the administration of GSE to animals prior to DOX treatment could play a cardioprotective role due to the inhibition of creatinine kinase activity [37]. In addition, such an experimental regime resulted in the decrease in DNA fragmentation in heart cells of mice exposed to DOX [37,38]. 

**Figure 1:**
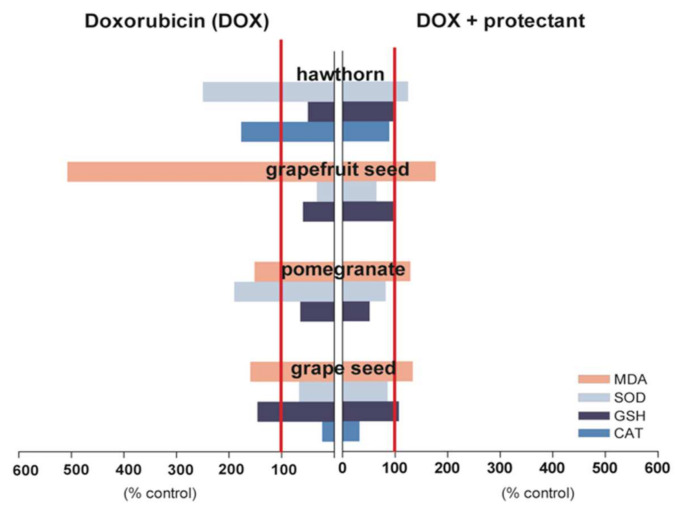
The oxidative-stress-associated cardiotoxic effects of DOX (left-hand side bars) are compared with cardioprotective activity of fruit extracts (right-hand side bars). Fruit extracts decreased MDA formation stimulated by DOX (control MDA values ranged from 7.92 to 182.65 nmol/g protein). The tested cardioprotectants restored the reduced by DOX level of GSH (control GSH values range was from 27.51 to 35.87 mg/g protein) as well as normalized activity of antioxidant enzymes SOD and CAT (100% CAT and SOD were in the range from 30.95 to 50.29 U/g protein and from 13.78 to 155.5 kU/mg protein, respectively).

The cardioprotective potential of grape seed proanthocyanins (GSP) was also observed in the experiments conducted by Ammar et al. [39], in which rats were treated with saline (1 mL/kg/day) for 9 days, DOX (15 mg/kg, *i.p.* as a single dose), GSP (70 mg/kg/day, orally for 9 days) or received GSP (70 mg/kg/day, orally) for 9 days and DOX (15 mg/kg, *i.p.*) on the 7th day 1 h after GSP administration. The study showed that GSP normalized serum biochemical markers of cardiotoxicity such as CK and LDH [39] as well as improved myocardial tissue antioxidant status (Figure 1). These experiments suggest the mechanism by which GSP ameliorate DOX-induced cardiotoxicity and it is mainly attributed to its antioxidative properties where GSP scavenged free radicals and blocked lipid peroxidation. The protective action of grapes was confirmed by the study on the effectiveness of wine polyphenolic compounds in mitigating DOX-induced cardiotoxicity by the Nrf2-dependent down-regulation of TGF-β regulation in rats. Namely, wine polyphenolic compounds improved the failing cardiac function and cardiac hypertrophy induced by DOX, reduced the extracellular matrix accumulation, and inhibited DOX-induced cardiac fibrosis, attenuated DOX-triggered oxidative stress and increased the levels of antioxidant enzyme activities, as well as inhibited DOX-induced mitochondria-dependent apoptosis [40].

#### 2.1.2. Pomegranate

Pomegranate (*Punica granatum* L.) is a nutrient-dense fruit rich in phytochemicals such as punicalagin, ellagitannins, anthocyanins, tannins, hydrolysable tannins, and punicic acid [41]. These phenolic compounds have attracted increasing attention as preventive agents for various oxidative-stress-related diseases. Therefore, pomegranate polyphenols could be a good candidate in preventing cardiotoxicity initiated by DOX. The cardioprotective effect of whole fruit extract of pomegranate (WFEP) was investigated in rat model [42]. Investigations included three groups of rats: the control (received water), DOX (received 10 mg/kg DOX through the femoral vein on day 16) and WFEP + DOX group (received 100 mg/kg/day WFEP orally for 18 days and on 16th day 10 mg/kg DOX).

This study revealed that the administration of whole fruit extract of pomegranate along with DOX restored QT interval, heart enzyme activity, markers of redox imbalance (Figure 1) and histopathological properties in rats to the control level and concluded that WFEP could exhibit cardioprotective properties.

#### 2.1.3. Hawthorn

Hawthorn (*Crataegus species*) has been used in folk medicine for centuries as a remedy, which treats cardiovascular diseases, cancer, diabetes, hyperlipidemia, and sexual weakness [43,44,45]. Flavonoids and procyanidins are the main active hawthorn compounds. The antioxidant properties of the aqueous extract of *Crataegus aronia* (CAE) in the context of protection against cardiotoxic effects of DOX were determined in albino Wistar rats [46]. Investigations included six groups of rats: control (received normal saline, for 28 days), CAE (were administered 200 mg/kg/d of CAE for 14 consecutive days, by oral gavage, then normal saline *i.p*. for the next 14 days), DOX (received accumulative dose of 15 mg/kg DOX in 6 equal *i.p*. injections (2.5 mg/kg every 2 days) over 14 consecutive days), DOX + CAE (received combined treatment of CAE and DOX for 14 consecutive days with the same doses and routes of administration as previously mentioned), CAE postDOX (were administered an accumulative dose of DOX (15 mg/kg) *i.p*. for the first 14 days, then a daily dose of CAE (200 mg/kg) orally for the next 14 days) and CAE preDOX (were administered a daily dose of *C. aronia* (200 mg/kg) for 14 days, then treated with an accumulative dose of DOX (15 mg/kg) *i.p*. for the next 14 days). The prevention and/or treatment of DOX-induced cardiotoxicity and heart failure were followed, and based on the obtained results, it was concluded that the administration of the aqueous extract of *C. aronia* with or following DOX augmented the endogenous antioxidant components of the rat heart and ameliorated DOX-induced cardiotoxicity (Figure 1).

#### 2.1.4. Grapefruit

Findings from epidemiological studies suggest an association between grapefruit (*Citrus paradisi L*.) consumption and reduced risk of coronary heart disease mortality [47]. In addition to the wide range of essential vitamins and minerals, grapefruit is rich in carotenoids and flavonoids. Since all these compounds are powerful antioxidants, ethanolic seed extract of grapefruit was assessed as an effective attenuator of DOX-induced oxidative stress in rat heart [48]. Adult male Sprague Dawley rats were divided into four groups: control (CON) was given peanut oil (vehicle), 0.5 mL daily by gastric gavage for 14 days; the second group received ethanol extract of grapefruit seed extract (GSE), 20 mg/kg/day by gastric gavage for 14 days; the third received DOX 20 mg/kg (*i.p*. single dose) following gastric gavage of GSE, 20 mg/kg/day for 14 days; the fourth and last group also had single dose *i.p.* DOX 20 mg/kg, but following the administration of peanut oil, 0.5 mL daily by gastric gavage for 14 days. The rats were sacrificed, and enzymatic and non-enzymatic cardiac antioxidants were determined (Figure 1). The results showed that pretreatment with GSE for 14 consecutive days caused the amelioration of all biochemical parameters altered by DOX, and such findings suggest a possible use of GSE in the prevention of DOX-induced cardiomiopathy.

### 2.2. Cardioprotective Potential of Vegetables

#### 2.2.1. Garlic

Garlic (*Allium sativum* L.) is a very popular vegetable that has been consumed since antiquity. Its regular intake reduces cholesterol, thereby decreasing the risk of cardiovascular diseases. Garlic is also known to exhibit antitumor, antimicrobial [49], and antioxidant [50] properties. Garlic co-administration was also shown to decrease DOX toxicity towards cardiac muscle cells [51]. The cardioprotective activity of garlic in rats treated with DOX was compared with that of probucol (PRO), which is a powerful antioxidant itself, but also increases the level of endogenous antioxidants [25]. In addition, by increasing the rate of LDL catabolism, this drug lowers the levels of cholesterol and triacyclglycerols in the bloodstream. Therefore, PRO is often used to protect heart cells during chemotherapy with anthracyclines [52]. In these studies, DOX (30 mg/kg *i.p.*) was administered in conjunction with PRO (cumulative dose 120 mg/kg administered in 12 doses for 30 days *i.p.*) or with garlic homogenate orally administered for 30 days at amounts of 250 mg/kg/day (G-250) or 500 mg/kg/day (G-500). The protective activity was assessed based on the measurements of oxidative stress biomarkers in rat hearts (Table 4).

The results demonstrated that the level of monitored markers in animals treated with DOX and garlic closely resembled the results obtained for the rat group treated with DOX and PRO. Hence, garlic can play a cardioprotective role, as it prevents DOX toxicity towards heart cells equally as effectively as the currently clinically used cardioprotectant.

#### 2.2.2. Tomato

Tomatoes (*Lycopersicon esculentum* L.) are popular diet components in many countries. This vegetable is a rich source of carotenoid—lycopene (Table 2)—as well as flavonol—quercetin—and vitamins A and C. Due to the fact that it reduces the properties of these phytochemicals, it was presumed that tomato extract and purified lycopene may ensure protection against cardiotoxicity stimulated by DOX. To verify this hypothesis, experiments were performed in which mice were treated with either DOX (15 mg/kg *i.p*.) or DOX together with tomato extract (1.2 g/kg or 2.4 g/kg administered *i.p.* 1 day before and 3 days after DOX treatment) or DOX with lycopene (1.7 mg/kg or 3.5 mg/kg administered *i.p*. 1 day before and 3 days after DOX treatment). CK activity was measured (Table 5) and the histopathological examination was executed [24].

**Figure 2:**
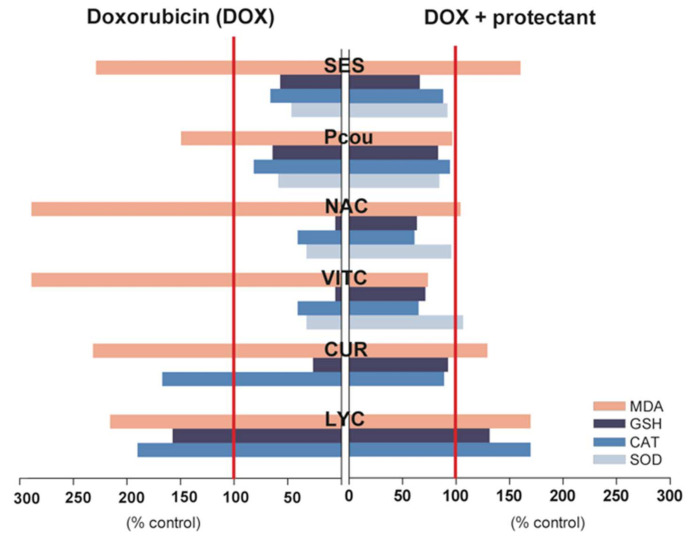
The oxidative-stress-associated cardiotoxic effects of DOX (left-hand side bars) are compared with cardioprotective activity of bioactive phytochemicals (right-hand side bars). Phytochemicals decreased MDA formation stimulated by DOX (control MDA values ranged from 57.7 to 1179 nmol/g protein). The tested cardioprotectants restored the level of GSH reduced by DOX (control GSH values range was from 0.01 to 99.8 μmol/g protein), as well as normalized activity of antioxidant enzymes SOD and CAT (100% CAT and SOD were in the range of 5.82 to 12.35 U/mg protein and from 1.10 to 8.69 U/mg protein, respectively).

The increased levels of CK were observed in animals treated with DOX. However, the results confirmed that tomato extract and lycopene co-administered with DOX to a significant degree decreased the activity of CK in a dose-dependent manner. In addition, the results of histopathological examination revealed that animal tissue treated with DOX together with tomato extract or lycopene at higher doses were characterized by the lower susceptibility of heart cells to necrosis compared to heart tissue sections from mice who were administered DOX only [24]. Alternative approaches have demonstrated the ability of lycopene to protect heart tissue affected by DOX [53]. During these experiments, rats were treated with DOX (10 mg/kg *i.p*.) or DOX with lycopene in two schemes. In the first, lycopene (4 mg/kg) was administered via indenter in corn oil for 10 days before treatment. In the second, lycopene was applied in the same way, but for 2 days before and 3 days after DOX treatment. The biochemical evaluation of heart sections demonstrated that the administration of lycopene during DOX therapy was particularly beneficial (Figure 2). Histopathological examination indicated, among other effects, a reduction in interstitial edema and degenerative states in the rats treated with lycopene and DOX as compared with hearts from rats treated with DOX only.

These results suggest that lycopene and tomato extract may protect the heart muscle against DOX toxicity [53]. According to Karimi et al. [24], such beneficial properties result from the neutralization of free radicals and inhibition of lipid peroxidation. In these studies, the administration of the popular food component was particularly valuable, indicating the potential of a proper diet to aid in the efficient protection of oncological patients from the toxic side effects of chemotherapy.

#### 2.2.3. Spinach

The wide variety of flavonoids in spinach (*Spinacia oleracea* L.) are responsible for the antioxidant properties of this vegetable [54]. The spinach phytocomplex was thus also proposed to be capable of preventing DOX toxicity of the heart muscle [55]. The mice were administered DOX (20 mg/kg *i.p*.) or DOX in conjunction with the spinach extract (ES) in a dose of 10 mg/kg/day. ES was given *i.p*. for 7 days before and 6 days after DOX treatment or only 6 days after DOX treatment. Table 6 summarizes the results of measurements regarding biochemical oxidative stress biomarkers.

The study confirmed the potential of spinach to protect heart cells from the toxic effects of DOX therapy. The histopathological observations and biochemical markers indicated that the most effective heart protection by spinach components occurred in animals that were administered ES before and after DOX treatment. In these animals, the lowest level of lipid peroxidation and the strongest stimulation of activity of antioxidant enzymes were observed [55]. 

#### 2.2.4. Beetroot

Beetroot (*Beta vulgaris* L.) is a rich source of betalains—water-soluble pigments containing heterocyclic nitrogen atoms in their structure. Purple red betacyanins and yellow betaxanthins belong to the group of betalains [56]. Betanin is a phytochemical occurring in beetroot in the highest concentration among all betalains. Other substances present in high concentrations in the peel of this vegetable are phenolic acids, e.g., p-coumaric acid and ferulic acid [57]. All these components display high antioxidant activity and were shown in vivo in mouse model to inhibit the development of skin and lung cancer. It is suggested that these beneficial properties resulted from the presence of betalains [58]. 

**Figure 3:**
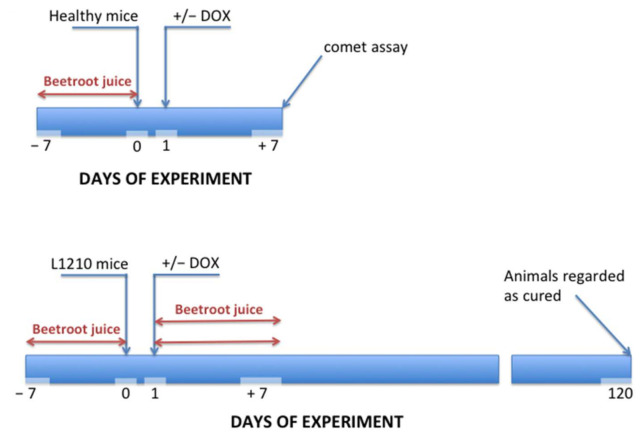
Experimental schemes for assessment of cardioprotective properties of beetroot tested in leukemia-bearing mice treated with DOX.

The cardioprotective properties of beetroot were tested in leukemia-bearing mice treated with DOX [59]. Two experimental schemes were tested (Figure 3). In the first, healthy mice were given beetroot juice to drink ad libitum instead of water for 7 days, then were administered a toxic dose of DOX (20 mg/kg *i.p.*) for 2 or 20 h. After this time, the level of DNA damage in cardiomyocytes was determined. The results of comet assay revealed very strong DNA fragmentation in the cardiomyocytes of the mice treated with DOX. This genotoxic effect was significantly decreased in the animal groups treated with anthracycline in conjunction with beetroot juice.

In the second scheme, mice bearing leukemia L1210 were tested. In these experiments, the animals were treated with either DOX (therapeutic dose of 7.5 mg/ kg *i.p.*), or beetroot juice, or DOX in conjunction with beetroot juice. Survival for 120 days was considered to indicate a complete cure. The results of these experiments indicated that treatment with DOX alone significantly prolonged the lifespan of leukemia-bearing mice; however, none of the animals reached the 120-day lifespan threshold. In contrast, in animal groups in which DOX administration was combined with beetroot juice, complete cures were observed in about 50% of the mice. These results suggest that simple dietary intervention might be a very effective means to protect oncological patients against undesirable effects of oxidative stress. After the completion of clinical studies, this intervention could be recommended to cancer patients undergoing chemotherapy.

### 2.3. Cardioprotective Potential of Medicinal Mushrooms

The cardioprotective role of medicinal mushrooms in DOX-induced cardiotoxicity has been evaluated for *Ganoderma lucidum* Karst (Reishi), a popular edible mushroom widely used for the general promotion of health and longevity in Asian countries. Several studies documented the anti-cancer potential of extracts and fractions of *G. lucidum* [60,61,62] and suggested their potential as an alternative therapy for breast and prostate cancer. *Ganoderma lucidum* extract (GLE) was also shown to be active in preventing DOX-associated cardiotoxicity, as determined by the monitoring of several cardiac toxicity biomarkers [63]. In addition, histological observations, hematology profiles, and electrocardiography parameters supported the protective effect of GLE against cardiotoxicity associated with DOX treatment [63]. Another study reported that davallialactone (DAVA) extracted from the mushroom *Inonotus xeranticus* (Bull.) Karst significantly increased the viability of DOX-injured H9c2 cells, and inhibited DOX-induced ROS production, apoptosis, and the expression of Cu/Zn SOD and Mn-SOD. DAVA also inhibited the expression of extracellular signal-regulated kinase (ERK) and c-Jun N-terminal kinase (JNK), which were activated by DOX. In an animal model, treatment involving DAVA significantly reduced cardiomyocyte lesions [64]. 

### 2.4. Cardioprotective Potential of Wheat

Wheat (*Triticum* species) is one of the most important cereal crops in the world. Eaten as a whole grain, it has been claimed to reduce the risk of cardiovascular disease, type 2 diabetes, and cancer due to its content containing unique health-promoting components, such as phenolic acids and flavonoids [65]. Considering their prophylactic actions against oxidative stress, the cardio-protective role of chemically standardized total polyphenolic extract of whole wheat grains (WWGPE) in DOX-induced cardiotoxicity was tested employing appropriate in vitro and in vivo preclinical assays [66]. Beneficial effects of wheat polyphenols on ROS accumulation, lipid peroxidation, protein carbonylation, endogenous redox systems as well as on intrinsic and extrinsic apoptotic signaling were proven in isolated rat cardiomyocytes exposed to DOX (1 μM). In vivo study was conducted on Wistar rats treated with distilled water containing 2% Tween 80, (*p.o.)* as the control. Purified forms of the most abundant phenolic components of the extract, i.e., ferulic acid (100 mg/kg, *p.o.)* or apigenin (100 mg/kg, *p.o*.) as reference or polyphenolic extract WWGPE (100 mg/kg, *p.o.)* were administered once daily for 7 consecutive days prior to DOX. DOX (3 mg/kg, *i.p.*) was administered on every alternative day for a total of three doses. According to such a study design, all findings obtained under in vitro conditions were confirmed in vivo, with an emphasis on the superior cardioprotective effect of WWGPE compared to ferulic acid and apigenin (Table 7).

### 2.5. Cardioprotective Potential of Herbs and Spices

#### 2.5.1. Ginkgo Biloba

Japanese ginkgo (*Ginkgo biloba* L.) is a unique tree that has been used in Chinese medicine for over 5000 years. Components found in its leaves, mainly flavonoids and terpenoids (e.g., ginkgolid B) are characterized by high antioxidant activity. In addition, ginkgo exhibits anti-angiogenic properties and influences gene expression in a way that may support cancer chemoprevention [67]. The administration of ginkgo extract during DOX therapy prevented the development of cardiomyopathy in rats [68]. Jasim [69] reported that ginkgo extract given to mice submitted to DOX treatment had the following beneficial effects on heart functioning: a decrease in lipid peroxidation in heart cells, the restored activity of enzymes involved in ROS neutralization to the normal level, the reversal of undesirable changes observed in ECG, and the occurrence of only minimal structural changes in the heart. These results supported the use of ginkgo extracts rich in flavonoids as a dietary supplement for limiting cardiotoxicity induced by DOX in oncological patients [68]. To corroborate the cardioprotective action of *G. biloba* (GB) against acute DOX-induced cardiotoxicity, another study was conducted on rats [69]. Within this study, rats were treated with distilled water, 5 mL/kg/day, orally for 10 days or with distilled water, 5 mL/kg, orally for 10 days and on the 8th day they received a single intra-peritoneal injection of DOX (20 mg/kg). The solution of GB (100 mg/kg/day) was applied over 10 days before and after DOX treatment. Changes in cardiac biomarkers due to the co-administration of GB with DOX are shown in Table 8.

#### 2.5.2. Red Sage

Red sage (*Salvia miltiorrhiza* Bunge) is a medicinal plant widely applied as a remedy in cardiovascular diseases in traditional Chinese medicine [70]. In herbology, its roots are mainly used. The major bioactive components of red sage are salvianic acids (SAs), including salvianic acid A, salvianic acid B, rosmarinic acid, and other phenolic acids. SAs belong to strong antioxidants that decrease the level of lipid peroxidation and display the ability to scavenge hydroxyl radicals [71]. A preparation of salvianic acids was tested to determine their cardioprotective properties in mice exposed to DOX. Animals were administered DOX only (15 mg/kg) or DOX in conjunction with SAs (40 mg/kg); the latter were administered to animals immediately after drug treatment and then for two consecutive days. The protective activity of SA preparation was assessed based on histological observations of heart tissue, changes in ECG, the measurements of antioxidant activity of phenolic acids, and CK activity. In groups of animals treated with DOX only, the formation of cytoplasmatic vacuoles, myofibrillar loss, and undesirable changes in ECG typical for DOX-induced cardiomyopathy occurred. The single DOX dose also increased the level of free radicals in heart muscles, MDA level, and CK activity. In contrast, the administration of DOX together with SAs resulted in a decrease in heart muscle damage, the normalization of ECG, and a decrease in MDA level and CK activity. These results suggest that SA preparation can ensure the protection of the heart during DOX therapy [72].

#### 2.5.3. Saffron

Saffron (*Crocus sativus* L.) has been frequently used as a spice and medicinal plant to promote human health for a long time. Its antioxidant activity originates from two bioactive compounds, crocin and safranal [73]. Saffron extract was shown to possess anti-inflammatory, anti-atherosclerotic, antigenotoxic, and cytotoxic activities [74,75]. Likewise, findings obtained from a study on DOX-treated rabbit hearts demonstrated that saffron extract improved the cardiomyocyte survival and function, preserved cardiac troponin T proteins, inhibited the p38 MAPK pathway, and activated the AKT/mTOR/4EBP1 pathway [76].

#### 2.5.4. Ginger

Ginger (*Zingiber officinale* Roscoe) is a spice widely used all over the world. It contains various chemical constituents, including phenols, terpenes, polysaccharides, lipids, organic acids, and raw fibers. The health benefits (antioxidant, anti-inflammatory, antimicrobial, and anticancer activity) of ginger are mainly attributed to its phenolic compounds, such as gingerols and shogaols [77]. As a very potent remedy, it could be of great interest in different cardioprotective aspects. Ajith et al. [78] followed the activity of ginger aqueous ethanol extract in the context of acute cardiac damage in rats caused by DOX. Protection was estimated by measuring the level of cardiac MDA, as well as AST and LDH activities. The obtained results concluded that ginger ameliorated DOX-induced cardiotoxicity [78].

#### 2.5.5. Phyllanthus urinaria

Plants of the genus *Phylanthus (Euphorbiceae*) originate from tropical and subtropical regions. They have long been used in Thai folk medicine to treat kidney and urinary bladder disturbances, intestinal infections, diabetes, and hepatitis B. One of the representatives is *Phyllanthus urinaria* L. (PU), a plant that is rich in alkaloids, flavonoids, lignans, phenols, and terpenes [79]. In extracts from this plant, the following compounds were identified: *N*-octadecan, β-sitosterole, ellagic acid, kaempferol, quercetin, gallic acid, rutin [80], corilagin, isostrictinin, geraniin [81] and others. Components of *Phyllanthus* were reported to protect the heart against DOX toxicity [25,82]. Specifically, the antioxidant activity of *PU* in the context of protection against cardiotoxic effects of DOX was determined in the heart myoblast cell line H9c2 [25]. The H9c2 cell line is derived from heart tissue of rat embryos. It retains several features characteristic of heart muscles and therefore is considered a good model of this tissue. During these studies, the cells were pre-incubated with ethanolic extract of PU (1 μg/mL or 10 μg/mL for 30 min). The cells were then treated with DOX (10^−9^–10^−5^ mol) and incubated for 48 h. The results of these experiments are shown in Table 9. The protective properties of PU extract were compared with those of vitamin C and *N*-acetylcysteine, whose antioxidant activity has been well documented (Figure 2).

The antioxidant activity of PU was higher than that of vitamin C or *N*-acetylcysteine, which suggests that extracts from this herb could be used to prevent cardiotoxicity induced by DOX. Additionally, during these studies, the cytotoxicity of combined therapy and the level of transcription factor NF-κB p50/p65 (marker of inflammation) were determined. It was observed that PU significantly decreased the cytotoxic effects of DOX in H9c2 cells, especially in combination with a higher concentration of this herb extract. The stimulation of NF-κB expression by DOX was fully inhibited by PU, which indicated yet a different mechanism of protection of H9c2 cells [25].

#### 2.5.6. Spirulina

Spirulina (*Spirulina platensis* (Gomont) Geitler) is a primitive blue–green seaweed characterized by a high content of nutrients and phytochemicals [83]. Due to its high content of protein, calcium, iron, and β-carotene, it is often used in the production of dietary supplements. Its biological activity, and above all its strong antioxidant and anti-inflammatory activity, can be attributed to compounds such as fatty acids (n−3, n−6), β-carotene, α-tocopherol, phycocyanin, and phenolic compounds [15]. Recent studies suggest that spirulina counteracts DOX-induced cardiotoxicity and, therefore, may improve the therapeutic index of this drug [84]. Khan [16] conducted experiments treating mice *i.p.* with either DOX (4 mg/kg/week for 4 weeks) or spirulina (250 mg/kg, orally, twice a day for 7 weeks), or DOX in conjunction with spirulina (250 mg/kg, orally, 3 days before DOX treatment and 7 days with DOX). The results of this study are shown in Table 10.

The combined DOX and spirulina treatment resulted in a significant reduction in the level of mouse heart muscle damage, such as the loss of myofibrils, the formation of vacuoles in the cytoplasm, and the enlargement of the mitochondria. In addition, it contributed to protection against lipid peroxidation, influenced restoring the proper activity of enzymes responsible for antioxidant protection, and reduced mortality in this group of mice. 

Khan [16] also tested spirulina for its potential cardioprotective effect towards DOX-treated Wistar rats. In their study, rats were treated with either DOX (2 mg/kg, intravenously for 5 days, followed by weekly once for 2 weeks) or DOX with spirulina (1000 mg/kg feed by enteral route for 7 days followed by the intravenous injection of DOX; spirulina supplementation was continued for 19 days) or DOX in conjunction with spirulina (250 mg/kg, orally, 3 days before DOX treatment and 7 days with DOX). The results of this research (Table 10) indicate the potential of spirulina supplements to reduce cardiotoxicity during DOX cancer chemotherapy.

### 2.6. Cardioprotective Dietary Supplement—CardiPro

CardiPro is an herbal preparation manufactured by Square Pharmaceuticals Ltd. (Bangladesh). It contains extracts from five herbs, 12.5% *Boerhaavia diffusa* L., 12.5% *Ocimum sanctum* L., 25% *Emblica officinalis* Gaertn., 25% *Withania somnifera* (L.) Dunal, and 25% *Terminalia arjuna* (Roxb.) Wight & Arn. Each of these herbs, tested separately, displayed certain biological properties with potential for protecting human organisms against cardiovascular diseases. Mohan [85] tested the effects of CardiPro on cardiotoxicity induced by DOX in mice. The mice were treated with DOX (four doses of 4 mg/kg *i.p.*), CardiPro (150 mg/kg, orally 2 times a day for 7 weeks), or DOX in combination with CardiPro (twice a day for 7 weeks simultaneously with the administration of DOX).

The cardiac muscle was examined for morphological changes, the presence of lipid peroxidation products, and the level of enzymes responsible for the disposal of free radicals (Table 11). All biomarkers suggested that despite the use of a non-toxic dose of DOX, some damage to the cardiac muscle was observed (the loss of myofibrils, the formation of vacuoles in the cytoplasm, a higher level of MDA, and a decrease in the activity of antioxidant enzymes). The histological lesions in the hearts of mice receiving DOX + CardiPro treatment were minimal compared to the hearts of the mice in the control group. 

CardiPro also contributed to the protection of mouse myocardium cells against lipid peroxidation, and antioxidative enzyme activity returned to normal. The researchers claimed that the study results demonstrate that CardiPro, a flavonoid-rich herbal preparation, can be used in the protection of myocardial cells against chronic toxicity induced by DOX [85].

### 2.7. Phytochemicals Isolated from Edible Plants with Cardioprotective Potential

#### 2.7.1. Sesamol

Sesamol is a natural phenolic compound found in sesame seeds (*Sesamum indicum* L.) and sesame oil. There is increasing body of evidence that shows sesamol may act as a metabolic regulator that possesses antioxidant, anti-mutagenic, anti-hepatotoxic, anti-inflammatory, anti-aging, and chemopreventive properties [86]. The modulator role that sesamol plays against oxidative stress through its radical scavenging ability and lipid peroxidation lowering potential was tested in rats in which cardiomyopathy was caused by DOX [87]. In this study, albino Wistar rats were randomly divided into four groups according to design: control—received normal saline (10 mL/kg body weight *p.o*.); SES—received sesamol 50 mg/kg body weight *i.p.* for 7 days and then alternatively with a vehicle for the next 2 weeks; DOX—treated with DOX (total cumulative dose of 15 mg/kg *i.p*. for 2 weeks; SES + DOX—pretreated with sesamol 50 mg/kg body weight *i.p.* for 7 days followed by DOX administration as in the DOX group. Sesamol protective action on myocardium was monitored by several biomarkers; some of them, such as changes of cardiac biomarkers and antioxidant status, are shown in Figure 2.

#### 2.7.2. p-Coumaric Acid

p-Coumaric acid (PC) is a phenolic phytochemical present in many edible plants (Table 2). The main sources of this compound are coffee, wine, chocolate, and beer. Due to its antioxidant activity, research was conducted on the ability of PC to protect the heart from oxidative stress caused by DOX. Rats received *i.p*. a single dose of DOX (15 mg/kg), or PC (100 mg/kg) orally for 5 days, or PC for 5 days followed by DOX. After 24 h from the end of feeding, the levels of selected cardiotoxicity biomarkers in the heart tissue were determined (Figure 2).

The administration of DOX alone contributed to a significant increase in the level of LDH, CPK, and MDA, whereas the administration of PC to animals for 5 days preceding the administration of anthracycline prevented this effect. The enzymatic activity of SOD and CAT, as well as the level of GSH in the group of animals treated with DOX alone, decreased significantly. In the case of rats that received PC before the administration of DOX, a smaller decrease in these activities was observed. In the group of rats treated with PC only, no significant changes were observed in the biomarker levels relative to the control group. Thus, PC is another candidate phytochemical that can play an important role in counteracting damage to the heart caused by oxidative stress induced by DOX therapy [88].

#### 2.7.3. Curcumin

Turmeric (*Curcuma longa* L.), a commonly used traditional mixture of Indian spices and food colorant, is a plant that exhibits cardioprotective properties [89]. This may be due to its ability to protect cells from oxidative stress, including the avoidance of lipid peroxidation. The main component of turmeric is diferuloyl methane, commonly named curcumin (Table 2). Curcumin and its analogues are characterized by a large variety of physiological and pharmacological effects [90]. These compounds are antioxidants with anti-inflammatory and anti-infective properties [89].

Venkatesan [90] tested the cardioprotective properties of curcumin in rats exposed to DOX. The group of animals was administered *i.p.* a single toxic dose of DOX (30 mg/kg), curcumin (*i.p.*), or DOX with curcumin (200 mg/kg 7 days before and 2 days after administration of DOX). After the experiments were completed, the level of conjugated dienes and MDA (main lipid peroxidation products), GSH content, and activity of GSH-Px and CAT in heart cells were determined. The results of the conducted biochemical tests are shown in Figure 2. These data indicate that the combination of DOX therapy with curcumin caused the cardiotoxicity markers to remain at control level [91]. Although curcumin was administered *i.p.*, these studies suggest that turmeric, particularly the curcumin present in it, could be used as a cardioprotective agent during chemotherapy administered orally in a form ensuring high bioavailability.

Curcumin effects against DOX-induced cardiotoxicity were also extensively investigated using a myocardial injury model in mice (in vivo) and rat cardiomyocytes (in vitro) [92]. The effects of administering curcumin an hour before DOX administration were evaluated by determining the heart rate and ECG’s ST segments, as well as LDH and CK activities in serum, caspase-3 activity, apoptosis rate, and histopathological changes of the myocardium (in vivo). Additionally, in vitro experiments were designated as follows: control group—cardiomyocytes were incubated under normal conditions; DOX group—cardiomyocytes were exposed to 1 μM DOX for 24 h; curcumin + DOX group: cardiomyocytes were treated with curcumin (5, 10, 20, or 40 μM) for 22 h prior to DOX exposure. SOD, GSH-Px and CAT activities along with MDA level, cell viability and the apoptosis rate were assessed to test ability of curcumin pretreatment to interact with DOX-induced cardiotoxicity [92].

Curcumin pretreatment resulted in a significant suppression of oxidative stress, which is one of the main components of toxicity provoked by DOX in rat cardiomyocytes.

#### 2.7.4. Chalcones

Chalcones (trans-1,3-diphenyl-2-propen-1-ones), widely distributed in edible plants, are precursors to all known flavonoids [93]. In in vitro studies, chalcones demonstrated antioxidant and anti-inflammatory properties [94]. These compounds are believed to regulate the activity of certain enzymes involved in DOX metabolism, e.g., aldo-keto reductases. These reductases catalyze the formation of secondary alcohol metabolites, primarily doxorubicinol (DOXol) generated after the administration of DOX or daunorubicinol (DNRol) arising after the administration of daunorubicin. Compounds such as DOXol and DNRol are postulated to contribute to the development of cardiotoxicity [26].

The influence of the chemical structure of chalcones on the activity of cytoplasmic reductases, which play an important role in the transformation of anthracyclines to cardiotoxic derivatives such as DOXol and DNRol, was studied with the use of cytosolic fraction from rabbit hearts and from human hearts. Twelve chalcones were examined, and their activity was compared with the activity of quercetin and other flavonoids (Table 12). The results provided evidence that chalcones, in particular 2’, 4’, and 2-trihydroxychalcone (Table 2), affected the level of anthracycline metabolites (DOXol and DNRol) in the isolated cytosols of rabbit and human hearts. Flavonoids, particularly quercetin, were demonstrated to be effective inhibitors of DOXol and DNRol formation in the case of rabbit cytosol, and DNRol inhibitors, but not DOXol inhibitors, in the case of human heart cytosol. In comparison, one of the most promising semi-synthetic flavonoids, monohydroxytlorutoside (monoHER), had no effect on formation inhibition of the above-mentioned secondary alcohol metabolites [95].

These studies demonstrate the potential of chalcones as effective inhibitors of aldehyde and carbonyl reductases in the heart. This is an important mechanism in protecting the heart against the formation of secondary alcohol metabolites, and thus against DOX- or DNR-induced cardiomyopathy [26].

#### 2.7.5. Catechins

Catechins (Table 2) are flavonoids with a high degree of prevalence, with green tea being a plant particularly rich in these compounds [96]. Catechins are known antioxidants, free radical scavengers, and iron chelators. In vivo, these compounds exhibited beneficial effects on the myocardium and prevented the development of atherosclerosis and cardiac hypertrophy [97]. In addition, green tea extract helped to maintain the appropriate cardiomyocytic architecture and their viability [98].

Given the above properties, the ability of catechin to prevent DOX-induced cardiotoxicity in rats was tested [99]. The animals were administered i.v. DOX (at a dose of 3 mg/kg/week), *i.p.* catechin (at a dose of 200 mg/kg/week), or catechin in combination with DOX in different schemes:

- A scheme of 20 mg/kg catechin, followed 30 min by DOX;
- A scheme of 10 mg/kg catechin 30 min before and 1 h after DOX treatment;
- A scheme of 200 mg/kg catechin, followed 30 min by DOX;
- A scheme of 100 mg/kg catechin 30 min before and 1 h after administration of DOX;
- A scheme of 500 mg/kg catechin, followed 30 min by DOX.

The experiments were carried out for one month; their results are summarized in Table 13.

The greatest reduction in DOX toxicity was observed for rat hearts after the administration of catechin at a dose of 20 mg/kg. However, during the same experimental cycle, an adverse effect of catechin on cardiac atrial contractility was demonstrated in rats receiving this compound only (200 mg/kg) [99]. Another study found that catechin-rich green tea could counteract the DOX-inducible modification of fatty acids in cardiomyocytes in vitro [96]. Thus, the data from preclinical studies suggest that catechins may prevent cardiotoxicity induced by DOX, yet further testing is necessary to determine whether they can be an effective component of a chemotherapy-supporting diet in oncological patients.

#### 2.7.6. Rutin

Rutin (rutinozide, Table 2) is a compound found in large quantities in buckwheat (*Fagopyrum esculentum* Moench). Significant amounts of this phytochemical also occur, among others, in elderberry, St. John’s wort, and sorrel. It has been suggested that rutin, due to its antioxidant properties, could have a beneficial impact on human health. In the context of chemotherapy with anthracyclines, the literature describes the effect of rutin on the pharmacokinetics of idarubicin (IDA; an anthracycline antibiotic used clinically) transformation into idarubicinol (IDol) in rat heart tissue. As with the anthracyclines discussed earlier, the formation of secondary alcohol metabolites, such as IDol, is an important factor responsible for IDA cardiotoxicity. During the performed tests, 0.5 mL of IDA (1 mg/mL) was pumped through the heart of the animals for 1 min in the absence or in the presence of rutin (10 μM). After completion of the experiments, IDA and IDol contents were determined using HPLC. The results of the conducted research demonstrated that in heart cells of animals exposed to IDA, rutin contributed to a significant reduction in the amount of IDol produced (up to approx. 60%) [29]. This phytochemical, however, did not affect the production of DOXol in the cytosol of human heart cells obtained after autopsy [26]. The results of the above studies indicate that rutin could be an effective cardioprotective factor during chemotherapy with the use of IDA. However, this compound does not seem to ensure the protection of cardiac tissue cells against the side effects of DOX.

#### 2.7.7. Caffeic Acid Phenylethyl Ester

Caffeic acid phenylethyl ester (CAPE, Table 2) is a biologically active agent found in propolis extract. This compound also occurs, among others, in pears, basil, thyme, tarragon, turmeric, rosemary, hawthorn, and coffee. CAPE is a phenolic compound with antioxidant properties [100]. Most likely owing to these properties, it was demonstrated that *i.p.* administration of CAPE at a dose of 10 μmoles/kg/day resulted in a reduction in cardiotoxicity in rats treated with a toxic dose of DOX (20 mg/kg). The animals were administered DOX *i.p.*, or DOX with CAPE (*i.p.*, 2 days before and 10 days after DOX treatment). The levels of relevant cardiotoxicity biomarkers were determined after 12 days of treatment (Table 14).

In the case of rats treated with CAPE + DOX, the activity of antioxidant enzymes was significantly increased (nearly doubled), which probably contributed to the protection of cardiac tissue against lipid peroxidation and protein oxidation (Table 14). In addition, the structure of mitochondria in heart cells of rats treated with DOX + CAPE was similar to the structure of mitochondria in control animal cells. The researchers concluded that CAPE significantly affected the inhibition of DOX cardiotoxicity [101].

#### 2.7.8. Oleuropein

Oleuropein is a polyphenolic compound that is present in high concentrations in olive oil and leaves of the olive tree (*Olea europaea* L.). It has attracted attention in recent years due to a variety of reported health benefits: cardioprotective, anti-inflammatory, antioxidant, anti-cancer, anti-angiogenic and neuroprotective functions. Beyond these actions, oleuropein has been reported to be capable of preventing DOX-induced cardiomyopathy [102]. Wistar rats with cardiotoxicity induced by DOX and treated with 1000 mg/kg or 2000 mg/kg of oleuropein (*i.p.*) showed preserved heart left ventricle contractility, attenuated inflammation and degenerative myocardial lesions, mitigated nitro-oxidative stress and the decreased expression of pro-apoptotic mediators and the derangement of myocardial metabolism.

#### 2.7.9. Cannabinoids

Cannabinoids (the active constituents of *Cannabis sativa* L.) and their derivatives have received considerable attention in recent years because of their extensive pharmacological properties. Cannabinoids were first developed as successful agents for alleviating chemotherapy-associated nausea and vomiting. Recent studies have revealed that cannabinoids have a wide range of therapeutic effects such as appetite stimulation, the inhibition of nausea and emesis, the suppression of chemotherapy or radiotherapy-associated bone loss, chemotherapy-induced nephrotoxicity and cardiotoxicity, pain relief, mood amelioration, and relief from insomnia [103].

Among the phyto cannabinoids, cannabidiol (CBD) is a major nonpsychoactive constituent of the plant *C. sativa* [104]. In a chronic model of DOX-induced cardiotoxicity in rats, CBD was demonstrated to suppress myocardial toxicity via attenuating oxidative stress, inflammation, and cell death pathways [105]. Recently, Hao et al. [106] demonstrated that CBD attenuated DOX-induced cardiotoxicity via augmenting mitochondrial biogenesis and the blunting of oxidative and nitrative stress and apoptosis. It is noteworthy that CBD also was shown to exert several cardioprotective actions against diabetic cardiovascular complications [107] and has been approved in Canada and Europe for the management of pain associated with multiple sclerosis. Cannabinoids’ function is mediated through CB1 and CB2 receptors and endogenous cannabinoid modulate their function. Interestingly, in rat myocytes, both CB1 and CB2 receptors are expressed. Endogenous cannabinoids in the ischemic condition exhibited a protective function via interacting with these receptors [108].

### 2.8. Other Cardioprotective Food Ingredients

#### 2.8.1. Selenium and Vitamins A, C, E, D

Numerous studies have demonstrated that certain micronutrients, such as selenium, vitamins A, C, and E administered orally, and vitamin D applied *i.p*., may also protect the heart against cardiotoxicity induced by DOX [109,110,111,112]. The administration of vitamin A prevented abnormalities in the hearts of rats exposed to DOX. It reduced the degree of lipid and protein damage in the heart cells and inhibited lactate dehydrogenase and creatine phosphokinase activity [110]. The administration of vitamin C to mice and guinea pigs protected against DOX-induced lipid peroxidation and led to a reduction in the risk of acute cardiotoxicity [113]. Vitamin E, administered to mice with DOX, decreased MDA levels with the simultaneous increase in protein content and GSH and SOD activity [113]. The systemic administration of vitamin D to rats, with DOX-induced cardiotomiopathy, caused a significant decrease in the levels of Troponin T, blood urea nitrogen and CK enzyme. This effect indicates that vitamin D can prevent damage to the rat myocardium and stabilize the membrane [112].

The literature is inconclusive regarding the cardioprotective effects of vitamins during chemotherapy in oncological patients, arousing controversy in the scientific community. Results of preclinical studies involving mice and clinical trials in oncological patients demonstrated that vitamin E protected the myocardium from the occurrence of acute cardiotoxicity but did not prevent the development of chronic cardiac toxicity induced by DOX [109]. Additionally, it was reported that vitamin E only slightly impacted heart protection during cancer treatment [114,115]. The currently held belief is that there is insufficient evidence supporting the effectiveness of the antioxidant vitamins as cardioprotective agents when administered together with anti-cancer drugs; thus, further studies are needed to resolve this issue [116]. 

Selenium has also been tested for its ability to protect the myocardium against the side effects of chemotherapy. Quiles [113] reported that the symptoms of DOX-induced cardiomyopathy were suppressed in rats given selenium orally at a dose of 2.5 mg/kg for 8 weeks. This micronutrient also inhibited the development of cardiotoxicity in rabbits. However, the protective effect of selenium in myocardia exposed to DOX has not been confirmed in studies conducted with dogs [111] or mice [113].

#### 2.8.2. Melatonin

Melatonin (5-methoxy-*N*-acetyltryptamine, Table 2), which is the regulator of the daily human sleep and waking cycle, is a hormone produced by the pineal gland. It also occurs in bacteria, protozoa, plants, fungi, invertebrates, and vertebrates [117]. In many edible plants (e.g., mustard seeds, walnuts and groundnuts, asparagus, tomatoes, fresh mint leaves, black tea), melatonin (MEL) occurs at even higher concentrations compared to the levels of this hormone in human blood at night [118]. It is believed that it plays an important role as an antioxidant, protecting biomolecules from damage caused by ROS [119,120,121].

Liu [31] tested MEL and its natural analogues, 6-hydroxymelatonin (6-OH MEL, Table 2) and 8-methoxy-2-propionamidetetraline (8-M-PDOT) for their effects on DOX-induced cardiotoxicity. Mice treated with DOX (25 mg/kg *i.p.*) and/or MEL, 6-OH-MEL, 8-M-PDOT were first used in the survival experiments. MEL and its analogues were administered orally at doses 10 mg/L of water 24 h before the administration of a single, toxic dose of DOX. Treatment with MEL and its analogues was continued for 5 days. After this time, the survival of the animals treated only with DOX and treated with DOX + 8-M-PDOT was 50%. In the remaining groups, the protective effect of MEL and 6-OH MEL against toxicity caused by DOX was observed [31]. To confirm the cardioprotective properties of MEL and 6-OH MEL, measurements were made to assess cardiac function in vivo. Mice were injected with a toxic dose of DOX (22.5 mg/kg *i.p.*) or saline (control group). MEL (0.5 mg in 0.1 mL of 10% alcohol) or 6-OH MEL (0.5 mg in 0.1 mL of 10% DMSO) were administered by a microosmotic pump (2.5 μg/h) 24 h before and for 5 days after treatment with DOX. The following parameters of the heart function were determined:

- Left ventricular diastolic pressure (LVEDP);
- Left ventricular systolic pressure (LVESP);
- the first derivative of left-ventricular pressure after time (± dP/dt);
- Ejection volume (SV);
- Cardiac efficiency (CO).

Results are summarized in Table 15.

The results indicate that MEL and 6-OH MEL contributed to the improvement of the contractile weakness caused by DOX [31]. There is substantial evidence that MEL is an important antioxidant and that it contributes to the protection of the heart cell membrane from lipid peroxidation, one of the factors responsible for the development of DOX-stimulated cardiotoxicity [112,122,123,124,125,126]. The protective effect of MEL is also explained by its contribution to increasing the level of GSH [123,126,127], the induction of SOD activity [125], the stimulation of CAT activity [127], and the inhibition of zinc level decline caused by DOX [122].

#### 2.8.3. CoQ10 and l-Carnitine

Coenzyme Q (Q) is a key lipophilic compound for cell bioenergetics and membrane antioxidant activities. It is an intracellular antioxidant that protects membrane phospholipids, mitochondrial membrane protein, and LDL-cholesterol from free radical-induced oxidative damage. It has been reported to be capable of recycling and regenerating other antioxidants such as vitamin C and vitamin E [128]. As a cofactor, it plays a crucial role in the mitochondria respiratory chain and ATP production [129].

l-carnitine is a non-protein amino acid (β-hydroxy-γ-trimethyl-amino-butyric acid) that is synthesized from the essential amino acids, lysine and methionine. It is able to scavenge free radicals, to protect antioxidant enzymes from oxidative damage, to maintain the efficiency of the mitochondrial electron transport chain, and even to stimulate the synthesis of antioxidant molecules, e.g., reduced GSH [130,131]. 

Considering above-mentioned facts, ameliorative potentials of CoQ10 or l-carnitine on DOX-induced cardiotoxicity in rats were hypothesized [132]. The verifying study was conducted on Wistar albino rats divided into six groups: control group; DOX group (10 mg/kg); CoQ10 group (200 mg/kg); l-carnitine group (100 mg/kg); DOX + CoQ10 group; DOX + l-carnitine group. Oral treatment of CoQ10 and l-carnitine started 5 days before a single dose of 10 mg/kg DOX that was injected intraperitoneally, then the treatment was continued for 10 days. Cardioprotection was investigated by measuring oxidative stress, inflammatory and heart parameters and CoQ10 and l-carnitine effects are presented in Table 16.

The obtained results pointed out that both pretreatments by CoQ10 or l-carnitine could restore measured parameters to control levels and suggested these compounds as good candidates for protection of the myocardium integrity in DOX induced toxicity.

### 2.9. Semi-Synthetic Compounds with Cardioprotective Properties

#### 2.9.1. MonoHER and Its Derivative Frederine

7-monohydroxyethylrutoside (monoHER) is a semi-synthetic flavonoid with antioxidant properties. Systematic studies demonstrated that monoHER protected mice against DOX-induced cardiotoxicity. The advantage of this compound was that it did not affect the anti-tumor activity of DOX. However, to achieve full protection against the cardiotoxicity of this anthracycline, it was necessary to use high doses of monoHER (500 mg/kg). Its cardioprotective properties probably resulted from the ability to neutralize free radicals and/or chelate iron ions implicated in ROS formation [133].

The results of early pre-clinical tests demonstrated DOX induced inflammation [134]. Therefore, monoHER was tested for anti-inflammatory properties in DOX-treated mice. As an indicator of the inflammation of cardiac cells, *N*-(carboxymethyl)lysine (CML) was used, an advanced glycation product formed in proteins by combined non-enzymatic glycoxidation reactions. The anti-inflammatory properties of monoHER were tested by determining the number of stained blood vessels containing CML and measuring the intensity of this color. In these experiments, animals were treated with DOX (4 mg/kg i.v.) or DOX in combination with monoHER (500 mg/kg i.v. 60 min before administration of DOX). The monoHER significantly reduced the level of CML, which suggests that it exhibits anti-inflammatory properties during DOX therapy [20].

A more recent study demonstrated that monoHER is a compound that is safe to administer to healthy volunteers (at dose 1500 mg/kg), indicating its potential for reducing the cardiac toxicity of DOX in treated cancer patients [135]. However, as noted, to achieve complete protection against DOX-stimulated cardiotoxicity, it is necessary to use high doses of monoHER. Taking monoHER as a lead structure, a series of new flavonoids was synthesized, including a compound named Frederine, with the highest antioxidant potential among the obtained derivatives [136]. It was then demonstrated that this semi-synthetic flavonoid (at a dose of 68 mg/kg administered to mice *i.p.* for 6 weeks) exhibited at least five times higher antioxidant activity than monoHER. Thus, in combination with DOX, may be an even better cardioprotective factor [133].

#### 2.9.2. Metformin

Metformin (MET) is a biguandine, synthesized in the 1920s from guanidine derived from French lilac (*Galega officinalis* L.) and it is one of the most used oral antihyperglycaemic drugs in the treatment of type 2 diabetes, taking its place in WHO’s Model Lists of Essential Medicines [137,138]. Another interesting property of MET is its beneficial impact to cardiovascular system, where MET-treated diabetes patients have less risk of myocardial infarction [139]. This feature led to further investigations of MET’s potential use in circumstances where cardiotoxicity was observed as a side effect of initial treatment, including radiotherapy or DOX chemotherapy [140,141]. 

Animal studies mostly used rats as a model and found cardioprotective properties of MET in DOX-treated animals. Alhowail and Almogbel [129] observed the better overall survival of DOX + MET-treated mice. Other studies found improvements in aortic flow and cardiac output, myocytes thickness, degeneration, and other histopathological features of heart tissue, interstitial inflammation, echocardiography parameters, and brain/heart weight restoration when rats were MET + DOX-treated, compared to DOX-treated animals. The results were accompanied with improvements in markers of membrane damage (LDH, CK-MB, and Troponin T), as well as in autophagy proteins (LC3B-II) [142,143,144,145,146]. Regarding the markers of oxidative stress, MET + DOX-treated rats showed a decrease in MDA (or TBARS) concentration and restored GSH concentration and SOD activity compared to DOX-treated animals, indicating that beneficial properties might be related to minimizing the effects of oxidative stress [144,145,146,147] As a result of molecular changes in MET + DOX-treated rats lower ratio of apoptosis and pro-apoptotic signaling (Caspase-3 activity, Bax/Bcl-2 ratio) contributing to the normal function of heart tissue [143]. 

Further mechanistic studies were carried out using rat and mouse cardiomyocytes to investigate the role of certain signaling pathways and physiological changes in MET-related cardioprotection from DOX treatment. Similarly, the studies found improvements in cell viability, particularly in apoptosis decrease in MET + DOX-treated cells. This effect was accompanied with the deregulation of pro-apoptotic signaling (Caspase-3, 8, and 9, Bax/Bcl-2 ratio, and cytochrome c release) [143]. Again, studies showed that MET + DOX-treated cardiomyocytes exhibited better oxidative status compared to DOX-treated cells. The ROS and Ca^2+^ concentrations were lower and the activity of CAT, GPx, and SOD activities improved in presence of MET [147]. DOX treatment of cardiomyocytes induced changes to AMPK activity, ferritin heavy chain (FHC), adiponectin and its receptor expression, mitochondrial activity, and NF-κB, ERK, and JNK signaling. However, DOX treatment in conjunction with MET reversed these molecular changes and restored cell phenotypes to exhibit properties more similar to unexposed controls [147]. Based on the pre-clinical results, further in vivo studies are expected that might initiate clinical trials for the prevention of DOX-induced cardiotoxicty by MET.

## 3. Strengths and Limitations

The current review represents a comprehensive database of natural compounds sharing a particularly important common feature—the inhibition of cardiotoxic side effects associated with the anthracycline treatment. Such a database represents a precious roadmap for further clinical studies (Table 17). There are several limitations and potential knowledge gaps present in this journey from bench to clinics. 

The first is the extrapolation of data gained in pre-clinical in vitro and animal studies to humans. Most of the results summarized in our review were gained in animal studies, but success in pre-clinical trials is usually not sustained during clinical trials, where less than 10% of candidate drugs become approved [148,149]. Such disproportion could be explained in differences in experimental design, inter-special differences, and dose translation. Together with improvements in animal welfare, ethics in science, the 3Rs principle, and the development of novel approaches in drug testing, we can expect the number of animals used for pre-clinical studies to decline. However, animal research remains essential in gaining information after all alternative in vitro and in silico methods are drained [150,151,152,153,154]. It is therefore important to promote basic research using up-to-date state-of-the art techniques and approaches to develop a candidate drug that will have cardiopreventive properties without lowering the antitumor properties of the drug and the efficacy of the therapy.

A second limitation of analyzing the comparative bioactivity of plant extracts is the question of extract standardization, which is particularly important for herbal medicines and is emphasized by regulatory bodies at the global level. For dietary supplements and other less regulated health promotion products, this question is not so crucial. Standardization was defined by the American Herbal Product association: “Standardization refers to the body of information and control necessary to product material of reasonable consistency. This achieved through minimizing the inherent variation of natural product composition through quality assurance practices applied to agricultural and manufacturing processes” [155]. If a principle active component is known, it is most logical to measure and quantitate proposed compound. This approach has certain limitations, since herbal extracts represent mixtures of many constituents that potentially all have different bioactivities/different activity strengths. Additionally, herbal raw material is prone to a lot of variation due to several factors, such as seasonal variation, ecotypic, genotypic and chemotypic properties, and drying and storage conditions. Hence, the standardization of plant extracts and potential herbal drugs is an overly complex issue that requires separate investigation and was not a part of this review. 

## 4. Conclusions and Future Perspectives

Anthracycline antibiotics, including doxorubicin, are amazingly effective anticancer drugs. However, their clinical use is limited by the dose-dependent development of cardiotoxicity. The possibility of rational dietary intervention with phytochemicals and/or supplementation with other natural ingredients to reduce cardiotoxicity is gaining increased attention. 

Nutritional interventions using plants with the appropriate composition of bioactive compounds should be more thoroughly studied as a potentially safe and effective way to prevent anthracycline-induced cardiotoxicity. The emphasis should also be focused to cancer patients with previous or active heart disease that might be at greater risk for unwanted side effects. Childhood cancer survivors are also at increased risk of cardiovascular complications in their adulthood [43]. A newly emerged challenge is the management of COVID-19 in cancer patients receiving cardiotoxic antineoplastics in whom deteriorated lung function may be responsible for many additional cardiac events [156]. Thus, prophylactic intervention is even more critical to prevent cardiotoxicity in this group.

However, it should be emphasized that very few studies have been conducted to date. In addition, there is no data documenting the inhibition of the development of cardiotoxicity (induced by anticancer drugs) by natural compounds in humans, and information available in the literature can be the basis for drawing contradictory conclusions. Hence, both pre-clinical and clinical trials in cancer patients are a major challenge in this area.

## Figures and Tables

**Table 1:** Biomarkers used in the determination of cardioprotective potential of compounds and plant extracts.

Biochemical Marker	Function	Changes in the Amount/Activity of Biomarker That Indicate the Increase of Cardiotoxicity
Aldehyde reductase	Catalyzes the formation of secondary metabolites of chemotherapeutics	↑
AST—aspartate transaminase	Catalyzes the reversible transfer of an α-amino group between aspartate and glutamate	↑
Caspase-3	Participates in cell apoptosis	↑
CAT—catalase	Catalyzes the decomposition of hydrogen peroxide, prevents the formation free radicals	↓
Carbonyl reductase	Catalyzes the formation of secondary metabolites of chemotherapeutics	↑
CK, CPK—creatine kinaze	Indicator of cardiomyocytes necrosis, catalyzes creatine phosphorylation to phosphocreatine which is an energy source for muscle cells	↑
CML—N^ε^-(carboxymethyl)lysine	Marker of cardiomyocytes inflammation formed during protein damage	↑
Conjugated dienes	Products of lipid peroxidation	↑
GSH—glutathione	Major cellular antioxidant	↓
GSH-Px—glutathione peroxidase	Catalyzes the decomposition of hydrogen peroxide by glutathione	↓
GST—glutathione S-transferases	Catalyze the conjugation of reduced glutathione to a wide range of substrates, usually resulting in detoxification	↓
LDH—lactate dehydrogenase	Participates in lipid peroxidation	↑
MDA and other substances reacting with thiobarbituric acid (TBARS)	Products of lipid peroxidation	↑
Myeloperoxidase	Catalyzes the oxidation of some carcinogens using hydrogen peroxide, plays an important role in their biotransformation	↑
NFκB p50/p65—transcription factor	Apoptosis inhibitor	↑
SOD—superoxide dismutase	Catalyzes dismutation of superoxide anion radical	↓
TNF-α—tumor necrosis factor	Cytotoxic for cancer cells	↑
Troponin I	Inhibitory subunit of the troponin complex, acting to inhibit actin-myosin interaction	↑

Arrow upwards—increase in cardiotoxicity biomarker, arrow downwards—decrease in cardiotoxicity biomarker.

**Table 2:** Structures of some phytochemicals with documented cardioprotective potential.

Compound	Structure	Ref.
Lycopene	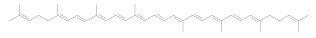	[24]
Curcumin	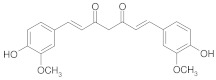	[25]
2′,4′,2-Trihydroxy chalcone	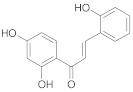	[26]
Catechin	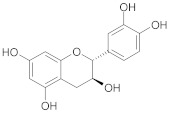	[27]
p-Coumaric acid	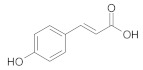	[28]
Rutin	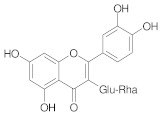	[29]
Caffeic acid phenylethyl ester	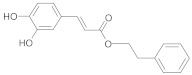	[30]
Melatonin	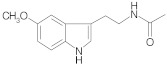	[31]

**Table 3:** Creatine kinase (CK) activity in mice treated with DOX in combination with grape seed extract (GSE) [37].

Group	% CK Activity ^a^
Control	100 ± 2.4
GSE	No change compared to the control
DOX	589.6 ± 60.7
GSE + DOX + GSE	142.4 ± 19.1

^a^ 100% CK activity = 1009 U/L.

**Table 4:** Levels of cardiotoxicity biomarkers in rats treated with DOX in combination with probucol (PRO) or garlic homogenate (G-250, G-500) [52].

Group	MDA Level [%] ^a^	GSH Level [%] ^b^	% CAT Activity ^c^	% SOD Activity ^d^	% GSH-Px Activity ^e^	TNF-α Expression
CON	100.0 ± 5.7	100.0 ± 8.5	100.0 ± 5.9	100.0 ± 11.5	100.0 ± 20.7	0.0
DOX	161.9±17.1	143.6 ± 2.5	27.9 ± 7.5	66.0 ± 7.7	43.0 ± 1.0	4.0
PRO + DOX	71.5 ± 6.5	105.9 ± 3.6	29.8 ± 4.0	90.3 ± 11.5	81.9 ± 15.5	1.6
G-250 + DOX	50.9 ± 8.5	93.4 ± 3.9	50.8 ± 6.7	88.4 ± 25.0	77.2 ± 15.5	2.8
G-500 + DOX	136.2 ± 9.7	131.4 ± 2.4	31.1 ± 6.5	110.6 ± 13.5	91.7 ± 5.2	2.9

^a^ 100% MDA = 203.03 nmol/g m.w.; ^b^ 100% GSH = 479.17 μg/g m.w.; ^c^ 100% CAT activity = 49.49 U/mg protein; ^d^ 100% SOD activity = 6.88 U/mg protein; ^e^ 100% activity GSH-Px = 0.193 U/mg protein.

**Table 5:** Effect of tomato extract (TE) and lycopene (LYC) on DOX-induced CK level changes in mice [24].

Group	% CK Activity ^a^
CON	100.0 ± 6.6
DOX	781.4 ± 99.2
TE (1.2 g/kg) + DOX + TE	425.5 ± 30.5
TE (2.4 g/kg) + DOX + TE	256.1 ± 24.0
LYC (1.7 mg/kg) + DOX + LYC	464.2 ± 33.2
LYC (3.5 mg/kg) + DOX + LYC	243.6 ± 25.9

^a^ 100% CK activity = 192.3 U/L.

**Table 6:** Effect of administering spinach extract (ES) on changes in the level of biomarkers of cardiotoxicity induced by DOX in mice model [55].

Group	MDA Level [%] ^a^	% CAT Activity ^b^	% SOD Activity ^c^	% GSH-Px Activity ^d^
CON	100.0 ± 10.3	100.0 ± 9.6	100.0 ± 13.4	100.0 ± 21.2
ES	96.0 ± 5.6	106.4 ± 11.7	118.0 ± 18.0	100.5 ± 5.4
DOX	148.0 ± 12.9	152.0 ± 15.9	143.6 ± 15.6	91.1 ± 12.6
ES + DOX + ES	111.5 ± 7.4	105.3 ± 8.5	196.4 ± 14.5	133.0 ± 14.1
DOX + ES	123.4 ± 11.5	128.7 ± 15.9	138.3 ± 18.0	100.2 ± 13.4

^a^ 100% MDA = 8.2 nmol/mg protein; ^b^ 100% CAT activity = 0.94 OD240 nmol/mg protein; ^c^ 100% Cu/Zn SOD activity = 20 U/mg protein; ^d^ 100% GSH-Px activity = 413 mU/mg protein.

**Table 7:** Effects of wheat polyphenols (polyphenolic extract of whole wheat grains—WWGPE; ferulic acid—FA; apigenin—API) on changes in biochemical parameters such as cardiotoxicity biomarkers induced by DOX in rat model [65].

Group	LDH Level [%] ^a^	CK Level [%] ^b^	AST Level [%] ^c^	Troponin I [%] ^d^	Troponin T [%] ^e^
CON	100.0 ± 9.9	100.0 ± 11.0	100.0 ± 11.2	100.0 ± 10.6	100.0 ± 10.6
FA	98.7 ± 10.6	98.8 ± 9.7	100.1 ± 10.3	100.0 ± 22.0	97.9 ± 12.8
API	98.3 ± 8.8	106.1 ± 11.2	97.9 ± 10.4	100.8 ± 12.9	100.0 ± 6.4
WWGPE	98.4 ± 9.9	103.5 ± 10.8	97.0 ± 10.7	102.3 ± 162.1	93.6 ± 6.4
DOX	129.9 ± 12.2	137.8 ± 13.6	135.9 ± 15.1	162.1 ± 17.4	142.5 ± 17.0
DOX + FA	112.4. ± 11.6	116.2 ± 11.9	115.8 ± 10.6	137.8 ± 14.4	121.3 ± 10.6
DOX + API	114.9 ± 11.9	119.0 ± 12.4	118.2 ± 11.0	135.6 ± 8.3	121.3 ± 14.9
DOX + WWGPE	108.3 ± 11.5	112.8 ± 10.2	112.6 ± 9.6	130.3 ± 11.4	110.6 ± 10.6

^a^ 100% LDH = 173.33 U/l; ^b^ 100% CK = 32.04 IU/mg protein; ^c^ 100% AST = 47.17 IU/l; ^d^ 100% Troponin I = 1.32 ng/mL; ^e^ 100% Troponin T = 0.47 pg/mL.

**Table 8:** Changes of cardiac biomarkers due to co-administration of *Ginkgo biloba* (GB) with doxorubicin in rats [69].

Group	MDA Level [%] ^a^	GSH Level [%] ^b^	Troponin I [%] ^c^	TNF-α Level [%] ^d^	Caspase-3 Level [%] ^e^
CON	100.0 ± 37.6	100.0 ± 15.9	100.0 ± 20.1	100.0 ± 47.9	100.0 ± 18.1
DOX	172.7 ± 67.3	58.5 ± 17.4	247.1 ± 44.3	161.1 ± 38.7	185.1 ± 34.4
GB + DOX + GB	145.4 ± 13.6	77.3 ± 13.4	192.9 ± 24.8	146.7 ± 16.5	153.0 ± 31.6

^a^ 100% MDA = 1.1 nmol/L; ^b^ 100% GSH = 24.83 pmol/L; ^c^ 100% Troponin I = 17 ng/L; ^d^ 100% TNF-α = 23.17 ng/L; ^e^ 100% caspase-3 = 13.33 pmol/L.

**Table 9:** The level of cardiotoxicity biomarkers in H9c2 cells treated with 1 μM DOX in combination with ethanolic *Phyllanthus urinaria* L. (PU) extract in various combinations [25].

Group	MDA Level [%] ^a^	GSH Level [%] ^b^	% SOD Activity ^c^	% CAT Activity ^d^
CON	100.0 ± 2.9	100.0 ± 2.3	100.0 ± 0.2	100.0 ± 4.0
PU 1 (1 µg/mL)	89.4 ± 1.8	213.5 ± 1.9	151.6 ± 2.2	136.8 ± 9.6
PU 10 (10 µg/mL)	88.5 ± 2.7	269.2 ± 3.8	170.7 ± 0.0	169.4 ± 12.4
DOX	289.0 ± 2.6	5.6 ± 3.4	32.6 ± 0.4	40.8 ± 1.5
PU 1 + DOX	55.6 ± 2.5	164.7 ± 0.8	147.3 ± 2.8	128.6 ± 8.5
PU 10 + DOX	30.7 ± 2.1	244.4 ± 7.1	160.9 ± 0.6	161.6 ± 11.7

^a^ 100% MDA = 1.179 nmol/mg protein; ^b^ 100% GSH = 0.266 nmol/mg protein; ^c^ 100% SOD activity = 5.817 U/mg protein; ^d^ 100% CAT activity = 11.854 nmol/min/mL/mg protein.

**Table 10:** The effect of DOX and spirulina on the level of cardiotoxicity markers in Wistar rats [16].

Group	MDA Level [%] ^a^	% SOD Activity ^b^	% GSH-Px Activity ^c^
CON	100.0 ± 13.9	100.0 ± 4.2	100.0 ± 7.1
DOX	167.4 ± 21.9	76.7 ± 8.2	72.6 ± 4.9
spirulina	101.6 ± 12.3	95.8 ± 3.7	102.7 ± 10.4
spirulina + DOX	138 ± 9.1	100.5 ± 6.3	92.8 ± 11.1

^a^ 100% MDA = 18.7 nmol/g heart tissue; ^b^ 100% SOD activity = 37.8 U/mg protein; ^c^ 100% GSH-Px activity = 56.6 nmol/mg protein.

**Table 11:** Effect of CardiPro on lipid peroxidation and the level of antioxidant enzymes in the heart tissue of DOX-treated mice (cumulative dose 16 mg/kg) [85].

Group	MDA Level [%] ^a^	% SOD Activity ^b^	% GSH-Px Activity ^c^
CON	100.0 ± 11.0	100.0 ± 9.3	100.0 ± 8.0
DOX	130.5 ± 3.7	79.5 ± 4.3	72.0 ± 6.4
CardiPro	79.3 ± 6.1	104.0 ± 2.7	106.0 ± 6.2
DOX + CardiPro	97.0 ± 11.0	101.3 ± 3.2	98.0 ± 3.6

^a^ 100% MDA = 16.4 nmol/g heart tissue; ^b^ 100% SOD activity = 37.5 U/mg protein; ^c^ 100% GSH-Px activity = 50 nmol/mg protein.

**Table 12:** Chalcone concentrations inhibiting by 50% (IC50) the formation of doxorubicinol (DOXol) and daunorubicinol (DNRol) in hearts of DOX- or DNR-treated rabbits [26].

Compound	IC_50_ [µM]	
	DOXol	DNRol
2′,4′,2-trihydroxychalcone	21.2 ± 3.6	33.8 ± 4.4
2′,4′,3-trihydroxychalcone	30.9 ± 5.5	83.5 ± 6.9
2′,4′,2,3- tetrahydroxychalcone	25.3 ± 5.6	39.9 ± 4.5
2′,4′,2,4-tetrahydroxychalcone	32.8 ± 8.8	59.1 ± 9.1
quercetin	13.7 ± 2.8	8.0 ± 2.0
morin	49.3 ± 6.3	18.8 ± 4.1

**Table 13:** Effect of catechin on body weight, heart weight, atrial shrinkage, and Q-T interval in rats treated with DOX at a dose of 3 mg/kg/week for one month [99].

Group	Body Weight [g]	Heart Weight [g]	Atrial Shrinkage d*f*/d*t* [g/s]	Q-T Interval [ms]
CON	245.0 ± 9.5	47.7 ± 1.3	18.0 ± 2.3	26.0 ± 1.0
catechin (200 mg/kg)	224.7 ± 5.3	40.0 ± 2.1	12.1 ± 1.4	26.1 ± 3.4
DOX	204.3 ± 60.1	34.1 ± 1.2	9.3 ± 3.4	40.4 ± 1.3
catechin + DOX (20 mg/kg)	241.7 ± 10.9	44.7 ± 1.2	14.1 ± 2.3	32.2 ± 3.3
catechin (10 mg/kg) + DOX + catechin	240.0 ± 8.6	43.5 ± 2.6	13.7 ± 2.1	33.2 ± 3.1
catechin (200 mg/kg) + DOX	235.0 ± 6.6	38.8 ± 3.1	13.2 ± 3.4	33.5 ± 2.2
catechin (100 mg/kg) + DOX + catechin	237.1 ± 5.4	38.6 ± 2.1	13.1 ± 2.3	35.2 ± 1.1
catechin (500 mg/kg) + DOX	219.2 ± 5.3	37.8 ± 3.1	10.5 ± 3.0	35.7 ± 4.4

**Table 14:** Effect of phenylethylene phenylethyl ester (CAPE) on the level of biomarkers of cardiotoxicity in the hearts of DOX treated rats [100].

Group	% CAT Activity ^a^	% SOD Activity ^b^	% GSH-Px Activity ^c^	MDA Level [%] ^d^	% Myeloperoxidase Activity ^e^	Content of Carbonyl Groups [%] ^f^
CON	100.0 ± 9.1	100.0 ± 10.2	100.0 ± 6.2	100.0 ± 10.1	100.0 ± 23.8	100.0 ± 27.3
DOX	168.8 ± 8.6	177.1 ± 12.1	116.4 ± 6.2	233.3 ± 13.8	238.0 ± 47.8	246.0 ± 37.4
CAPE + DOX + CAPE	207.0 ± 10.8	192.4 ± 12.1	166.7 ± 6.7	130.8± 8.7	131.2 ± 14.5	112.2 ± 33.8

^a^ 100% CAT activity = 0.186 U/g protein; ^b^ 100% SOD activity = 0.157 U/mg protein; ^c^ 100% GSH-Px activity = 2.535 U/g protein; ^d^ 100% MDA = 25.16 nmol/g tissue; ^e^ 100% myeloperoxidase activity = 1.207 mU/g protein; ^f^ 100% content of carbonyl groups = 0.139 nmol/mg protein.

**Table 15:** Effect of melatonin (MEL) and 6-OH MEL on cardiac function in DOX-treated mice [31].

Group	LVEDP [mmHg]	LVESP [mmHg]	+dP/dt [mmHg/s]	−dP/dt [mmHg/s]	SV [µL]	CO [mL/min]
CON	4.8 ± 0.7	64.7 ± 3.8	2638 ± 127	2010 ± 248	9.7 ± 0.5	4.3 ± 0.5
DOX	10.7 ± 2.1	37.3 ± 5.5	904 ± 156	727 ± 153	4.1 ± 0.4	1.6 ± 0.3
MEL	3.0 ± 0.8	63.2 ± 3.2	2664 ± 120	1978 ± 163	9.2 ± 0.7	4.2 ± 0.6
MEL + DOX + MEL	4.3 ± 0.8	60.2 ± 1.2	1914 ± 95	1629 ± 143	7.4 ± 0.8	3.2 ± 0.4
6-OH MEL	4.5 ± 1.1	63.9 ± 4.4	2384 ± 334	1863 ± 299	9.5 ± 0.6	4.1 ± 0.5
6-OH MEL + DOX + 6-OH MEL	5.8 ± 0.5	57.0 ± 5.2	1774 ± 208	1586 ± 121	7.7 ± 0.6	3.0 ± 0.5

**Table 16:** Changes of oxidative stress parameters, inflammatory cytokine, and cardiac markers in rats due to administration of CoQ10 or l-carnitine [132].

Group	% MDA Level ^a^	% GSH Level ^b^	% TNF-α Level ^c^	% LDH Level ^d^
CON	100.0 ± 24.5	100.0 ± 17.9	100.0 ± 11.1	100.0 ± 12.1
DOX	555.9 ± 80.2	77.3 ± 16.0	491.9 ± 30.9	400.2 ± 37.5
CoQ10	145.6 ± 14.1	118.9 ± 10.4	133.8 ± 56.6	95.4 ± 18.0
l-car	161.9 ± 19.0	123.6 ± 5.7	92.4 ± 9.8	104.2 ± 17.8
CoQ10 + DOX	345.5 ± 63.0	108.5 ± 9.4	179.5 ± 16.2	130.5 ± 18.2
l-car + DOX	347.7 ± 90.9	101.9 ± 7.5	282.3 ± 19.6	181.3 ± 15.4

^a^ 100% MDA = 11.79 nM/g; ^b^ 100% GSH = 1.06 µM/g; ^c^ 100% TNF-α = 40.25 pg/mL; ^d^ 100% LDH = 100.80 U/mL.

**Table 17:** Cardioprotective mechanisms of natural products and synthetic compounds.

Plant/Compound	Markers of Oxidative Stress	Markers of Membrane Damage	Antiinflammatory Properties	Heart Cell Viability	Histopathological Features of Heart Tissue	Cardiac Function	Myocardial Infarction	Mortality
Grapes	↓	↓		↑				
Pomegranate	↓				↓	↑		
Grapefruit	↓							
Hawthorn	↓							↓
Garlic	↓	↓			↓			
Tomato	↓	↓		↑	↓			
Spinach	↓	↓			↓			
Beetroot	↓							↓
*G. lucidum*	↓			↑	↓	↑		
Wheat	↓	↓		↑				
*G. biloba*	↓	↓		↑	↓	↑	↓	
Red sage	↓	↓			↓	↑		
Ginger	↓	↓	↑	↑				
Saffron	↓	↓	↑	↑				
*P. urinaria*	↓		↑					
Spirulina	↓	↓	↑		↓			↓
Seasamol	↓	↓						
Coumaric acid	↓	↓						
CAPE	↓	↓		↑	↓	↑		
Oleuropein	↓		↑	↑		↑	↓	
Cannabinoids	↓		↑			↑		
Vitamin C	↓	↓	↑					
Vitamin A	↓	↓						
Vitamin E	↓	↓						
Vitamin D	↓	↓		↑				
Curcumin	↓	↓	↑		↓	↑	↓	
Catechins	↓	↓		↑		↑		↓
Melatonin	↓	↓				↑		
CoQ10	↓		↑			↑		
l-carnitine	↓		↑			↑		
Cardi-pro	↓	↓			↓		↓	
MonoHER	↓		↑					
Metformin	↓	↓	↑	↑	↓	↑	↓	

Arrow downwards—decrease in the marker/property value; arrow upwards—increase in the marker/property

## Data Availability

Not applicable.
